# Interventricular septal thickness on cardiac computed tomography as a novel risk factor for conduction disturbances in patients undergoing transcatheter aortic valve replacement

**DOI:** 10.1093/europace/euae113

**Published:** 2024-05-01

**Authors:** Nili Schamroth Pravda, Yonatan Shaleve, Ygal Plakht, Gideon Shafir, Tzil Grinberg, Maya Wiessman, Yaron Aviv, Hana Vaknin Assa, Pablo Codner, Gregory Golovchiner, Alon Barsheshet, Ran Kornowski, Arthur Shiyovich, Ashraf Hamdan

**Affiliations:** Department of Cardiology, Rabin Medical Center, 39 Jabotinsky Street, Petach Tikva 49414, Israel; Faculty of Medicine, Tel Aviv University, P.O.B 39040 Ramat Aviv, Tel Aviv 69978, Israel; Faculty of Medicine, Tel Aviv University, P.O.B 39040 Ramat Aviv, Tel Aviv 69978, Israel; Internal Medicine ‘F’ (Recanati), Rabin Medical Center, Petach Tikva, Israel; Department of Nursing, Faculty of Health Sciences, Ben-Gurion University of the Negev, and Department of Emergency Medicine, Soroka University Medical Center, Beer-Sheva, Israel; Faculty of Medicine, Tel Aviv University, P.O.B 39040 Ramat Aviv, Tel Aviv 69978, Israel; Department of Radiology, Rabin Medical Center, Petach Tikva, Israel; Department of Cardiology, Rabin Medical Center, 39 Jabotinsky Street, Petach Tikva 49414, Israel; Faculty of Medicine, Tel Aviv University, P.O.B 39040 Ramat Aviv, Tel Aviv 69978, Israel; Department of Cardiology, Rabin Medical Center, 39 Jabotinsky Street, Petach Tikva 49414, Israel; Faculty of Medicine, Tel Aviv University, P.O.B 39040 Ramat Aviv, Tel Aviv 69978, Israel; Department of Cardiology, Rabin Medical Center, 39 Jabotinsky Street, Petach Tikva 49414, Israel; Faculty of Medicine, Tel Aviv University, P.O.B 39040 Ramat Aviv, Tel Aviv 69978, Israel; Department of Cardiology, Rabin Medical Center, 39 Jabotinsky Street, Petach Tikva 49414, Israel; Faculty of Medicine, Tel Aviv University, P.O.B 39040 Ramat Aviv, Tel Aviv 69978, Israel; Department of Cardiology, Rabin Medical Center, 39 Jabotinsky Street, Petach Tikva 49414, Israel; Faculty of Medicine, Tel Aviv University, P.O.B 39040 Ramat Aviv, Tel Aviv 69978, Israel; Department of Cardiology, Rabin Medical Center, 39 Jabotinsky Street, Petach Tikva 49414, Israel; Faculty of Medicine, Tel Aviv University, P.O.B 39040 Ramat Aviv, Tel Aviv 69978, Israel; Department of Cardiology, Rabin Medical Center, 39 Jabotinsky Street, Petach Tikva 49414, Israel; Faculty of Medicine, Tel Aviv University, P.O.B 39040 Ramat Aviv, Tel Aviv 69978, Israel; Department of Cardiology, Rabin Medical Center, 39 Jabotinsky Street, Petach Tikva 49414, Israel; Faculty of Medicine, Tel Aviv University, P.O.B 39040 Ramat Aviv, Tel Aviv 69978, Israel; Department of Cardiology, Rabin Medical Center, 39 Jabotinsky Street, Petach Tikva 49414, Israel; Faculty of Medicine, Tel Aviv University, P.O.B 39040 Ramat Aviv, Tel Aviv 69978, Israel; Division of Cardiovascular Medicine, Department of Medicine, Brigham and Women’s Hospital, Harvard Medical School, Boston, MA 02115, USA; Department of Cardiology, Rabin Medical Center, 39 Jabotinsky Street, Petach Tikva 49414, Israel; Faculty of Medicine, Tel Aviv University, P.O.B 39040 Ramat Aviv, Tel Aviv 69978, Israel

**Keywords:** Interventricular septum, TAVR, Conduction disturbances

## Abstract

**Aims:**

We examined whether thickness of the basal muscular interventricular septum (IVS), as measured by pre-procedural computed tomography (CT), could be used to identify the risk of conduction disturbances following transcatheter aortic valve replacement (TAVR). The IVS is a pivotal region of the electrical conduction system of the heart where the atrioventricular conduction axis is located.

**Methods and results:**

Included were 78 patients with severe aortic stenosis who underwent CT imaging prior to TAVR. The thickness of muscular IVS was measured in the coronal view, in systolic phases, at 1, 2, 5, and 10 mm below the membranous septum (MS). The primary endpoint was a composite of conduction disturbance following TAVR. Conduction disturbances occurred in 24 out of 78 patients (30.8%). Those with conduction disturbances were significantly more likely to have a thinner IVS than those without conduction disturbances at every measured IVS level (2.98 ± 0.52 mm vs. 3.38 ± 0.52 mm, 4.10 ± 1.02 mm vs. 4.65 ± 0.78 mm, 6.11 ± 1.12 mm vs. 6.88 ± 1.03 mm, and 9.72 ± 1.95 mm vs. 10.70 ± 1.55 mm for 1, 2, 5 and 10 mm below MS, respectively, *P* < 0.05 for all). Multivariable logistic regression analysis showed that pre-procedural IVS thickness (<4 mm at 2 mm below the MS) was a significant independent predictor of post-procedural conduction disturbance (adjOR 7.387, 95% CI: 2.003–27.244, *P* = 0.003).

**Conclusion:**

Pre-procedural CT assessment of basal IVS thickness is a novel predictive marker for the risk of conduction disturbances following TAVR. The IVS thickness potentially acts as an anatomical barrier protecting the underlying conduction system from mechanical compression during TAVR.

## Graphical Abstract

**Graphical Abstract:**
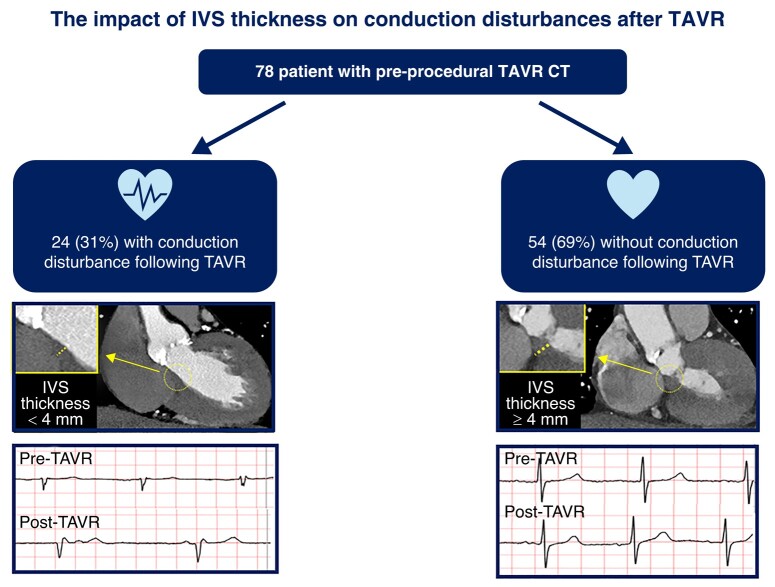

**What’s new?**

- Pre-procedural CT assessment of basal muscular interventricular septum (IVS) thickness is a novel predictive marker for the risk of conduction disturbances following transcatheter aortic valve replacement (TAVR).
- Patients with thinner IVS (<4 mm at 2 mm below the membranous septum) had a seven times higher risk of post-TAVR conduction disturbance.
- Our findings suggest that the IVS acts as an anatomical barrier protecting the underlying conduction system from mechanical compression during TAVR.

## Introduction

Transcatheter aortic valve replacement (TAVR) has become the guideline-directed standard of treatment in high-risk surgical patients and an accepted alternative to surgery in lower risk patients.^1,2^ One of the major complications of TAVR is the risk of conduction disturbances and the ensuing need for a permanent pacemaker (PPM). The reported rates of PPM following TAVR range from 3.4–25.9% and have been shown to be associated with morbidity and mortality.^1–3^ The most frequent conduction disturbance following TAVR is new onset left bundle branch block (LBBB) with reported rates ranging from 4–65%.^4^ New onset LBBB is not benign and necessitates extended monitoring as this can progress into complete atrioventricular block with the ensuing need for PPM insertion, which may occur in 3.4–25.9% of patients undergoing TAVR.^3^ There are multiple demographic (older age, male sex), anatomic [extent of calcification, membranous septum (MS) length, higher mean aortic valve gradient], electrical (pre-procedural right bundle branch block), and procedural risk factors (self-expanding valve, deeper valve implantation, balloon post-dilation) associated with conduction disturbances (CD) following TAVR.^3^ The main mechanism of CD following TAVR is a product of the implanted valve being inserted into the region of the aortic annulus and close to the conduction system. The conduction system traverses this region with the Bundle of His located at the base of the muscular interventricular septum (IVS). We hypothesized that the basal muscular IVS may act as a protective anatomical cushion against the mechanical forces of the TAVR devices on the underlying conduction system. In the present study, we examined whether the thickness of the basal IVS as measured by pre-procedural computed tomography (CT) could be used as a risk marker for conduction disturbances occurring following TAVR.

## Methods

### Study population

One hundred seventy-five consecutive patients with symptomatic severe aortic stenosis were evaluated with CT before TAVR between 2016 and 2018. The excluded patients and reasons for this are shown in Supplementary material online, *Figure S1*. Seventy-eight patients represented the final study population.

### CT acquisition protocol

All patients underwent contrast-enhanced CT using a 256-slice system (Brilliance iCT, Philips Healthcare, Cleveland, OH, USA) before TAVR. The contrast-enhanced CT scan was acquired with a collimation of 96 × 0.625 mm and a gantry rotation time of 330 ms. Tube current was 485 mA at 100 kV, pitch value was 0.2, and scan direction was cranio-caudal. Intravenous injection of 70–85 mL of non-ionic contrast agent (Iopromide 370, Bayer Shering, Berlin, Germany) at a flow rate of 3.5 mL/s was followed by a 30 mL saline chase bolus (5 mL/s). Automated peak enhancement detection in the descending aorta was used for timing of the scan, and the data acquisition was automatically initiated at a threshold level of 100 Hounsfield units. Acquisition was performed during an inspiratory breath-hold while the electrocardiogram (ECG) was recorded simultaneously to allow retrospective gating of the data. All images were reconstructed with a slice thickness of 0.67 mm and a slice increment of 0.34 mm.

### CT data analysis

The contrast-enhanced CT datasets were transmitted to a dedicated CT workstation (Philips, IntelliSpace Portal, version 11) to allow for multiplanar reformations. The contrast-enhanced CT data were reconstructed at 10% increments of the RR-interval and the peak systolic phase was used for data analysis.

**Figure 1:**
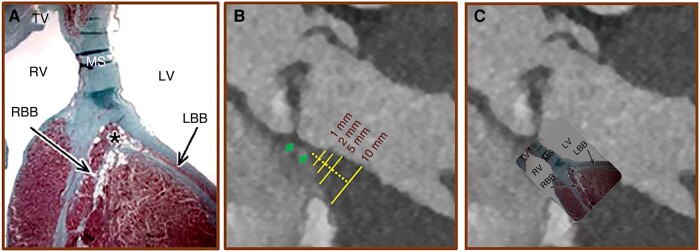
Schematic illustration of the conduction system and CT based measurement of the basal muscular part of the IVS. (*A*) Illustration of the conduction system. (*B*) CT coronal view demonstrating how the thickness of the muscular part of the IVS was measured at 1, 2, 5, and 10 mm below the membranous septum (green arrows). (*C*) CT coronal view with superimposed illustration of the anatomy of the conduction system. Since the conduction axis is located within the basal IVS, the thickness of this region may serve protect against conduction disturbances following TAVR. LBB, left bundle branch; MS, membranous septum; RBB, right bundle branch; TV, tricuspid valve; TAVR, transcatheter aortic valve replacement. *Bundle of His. Adapted from Anatomical Considerations for His Bundle Pacing. Circulation: Arrhythmia and Electrophysiology. 2019; 12:e006897, used with permission from Wolters Kluwer Health, Inc.

Membranous septum length was measured in the systolic non-reformatted standard coronal plane and was defined by the distance from the basal aortic annulus at the level of the intersect of the right and non-coronary cusp and the transition of the membranous to the muscular part of the IVS as previously described from our group.^5^ The thickness of the muscular part of the IVS was measured in the same projection at 1, 2, 5, and 10 mm below the MS (*Figure 1*). The observer, who was blinded to clinical and outcomes data, was initially trained before performing the measurements.

The aortic annulus dimensions were measured after reconstruction of the aortic annulus from the sagittal and the coronal planes using double oblique multiplane reformations, as described previously.^6^

### Transcatheter aortic valve replacement

Prostheses used in the study included Medtronic CoreValve and Evolute (Medtronic, Minneapolis, MN) in 39 patients (50.0%), Lotus valve (Boston Scientific) in 2 patients (2.6%), and Sapien Edwards (Edwards Lifesciences) in 37 patients (47.4%). Size selection was based on CT perimeter measurements for self-expandable valve and on area for balloon expandable valve. The TAVR procedure at our institution has been described previously.^7^

Implantation depth was determined fluoroscopically in the implantation projection, pre-determined using CT to verify orthogonality with respect to the aortic annulus. Implantation depth was defined as the average distance from the native aortic annulus plane (on the side of the non-coronary or right cusp and on the side of the left coronary cusp), to the most proximal edge of the implanted valve (deepest level in the left ventricle).

### Data collection

Demographic, clinical, imaging, and procedural data were collected retrospectively from our centre’s prospective ongoing TAVR database. The assessment and selection process of patients with symptomatic severe aortic stenosis at our centre have been previously described.^8^ All patients were discussed in a dedicated multidisciplinary Heart Team forum including interventional cardiologists, cardiac imaging specialists, cardiac surgeons, and geriatricians if needed. All patients were monitored for at least 24 h in the cardiac intensive care unit following the intervention with continuous telemetry monitoring and serial ECG during admission.

### Follow-up and study outcomes

The primary composite outcome was defined as conduction disturbance following TAVR during hospitalization, which was defined as: Mobitz Type II atrioventricular block, complete atrioventricular block, LBBB, or PPM implantation.

In all cases of conduction disturbances, our local practice is consultation with the electrophysiology team, who follow the instruction of the European Society of Cardiology guidelines.^3^ Patients with high degree atrioventricular block, new alternating bundle branch block, and patients with new conduction disturbance on the background of right bundle branch block are referred for PPM insertion.^3^ In cases of new onset LBBB, all patients have extended inpatient monitoring for dynamic ECG changes and are discussed in a multidisciplinary forum.^3^

This research was approved by the local ethics committee.

### Statistical analysis

Statistical analysis was performed using IBM SPSS 29 (SPSS Inc., Chicago, IL, USA) software. Patient characteristics were presented as mean and standard deviation (SD) for continuous variables and numbers (*n*) and per cent (%) for the categorical data. Comparison of baseline data and variables of IVS thickness between the groups with and without the primary outcome was done using the χ^2^ test and Fisher’s exact test for categorical variables and Student’s *t*-test for count variables. Logistic regression at a univariate level was done to examine the relationship between the IVS thickness and the outcome. In addition, the construction of a receiver operating characteristic (ROC) curve was done with the calculation of the *c*-statistic value. Youden’s index was used in conjunction with ROC analysis. The maximum value of the index was used as a criterion for selecting the optimum cut-off point. Multivariate analysis included the construction of a logistic regression model that included the thickness parameter with the highest discriminative ability. Baseline variables of clinical importance and/or with a statistically significant relationship with the outcome at a univariate level were also included in the model. The discriminative ability of the final model (that included the parameter of IVS thickness) compared to the model that contained only baseline variables was tested using the net reclassification improvement calculation. For each test, a *P* < 0.05 is considered statistically significant. Inter- and intra-observer variability was tested at 1, 2, 5, and 10 mm below the MS in 15 randomly selected patients according to the Bland and Altman method. In addition, interclass correlation coefficient was determined.

## Results

Our study included 78 patients, and *Table 1* shows the baseline characteristics of the cohort. The average age was 76.6 ± 8.7 years, 50% were male. A self-expanding valve was used in half of the cohort (51.3%). In terms of ECG characteristics prior to the TAVR procedure, 10.3% of the cohort had prior RBBB and 17.9% had atrial fibrillation. *Table 2* shows CT and anatomical parameters of the cohort.

**Table 1:** Baseline characteristics of the cohort and subgroup according to primary outcome

Parameter	Without conduction defect post-TAVR	With conduction defect post-TAVR	Total *n* = 78	*P* value
	*n* = 54	*n* = 24		
Demographics				
Sex (male)	23 (42.6)	16 (66.7)	39 (50.0)	0.050
Age (years)	75.70 (9.15)	78.46 (7.55)	76.55 (8.74)	0.200
Comorbidities				
Diabetes	16 (29.6)	9 (37.5)	25 (32.1)	0.492
Hyperlipidaemia	35 (64.8)	13 (54.2)	48 (61.5)	0.372
Hypertension	42 (77.8)	21 (87.5)	63 (80.8)	0.370
Coronary artery disease	19 (35.2)	9 (37.5)	28 (35.9)	0.844
Previous coronary artery bypass surgery	7 (13.0)	4 (16.7)	11 (14.1)	0.729
Previous percutaneous coronary intervention	12 (22.2)	7 (29.2)	19 (24.4)	0.510
Previous myocardial infarction	7 (13.0)	5 (20.8)	12 (15.4)	0.498
Pre-operative ECG				
Atrial fibrillation	12 (22.2)	2 (8.3)	14 (17.9)	0.205
Right bundle branch block	5 (9.3)	3 (12.5)	8 (10.3)	0.696
Left bundle branch block	6 (11.1)	0	6 (7.7)	0.169
First degree atrioventricular block	1 (1.9)	4 (16.7)	5 (6.4)	0.029

Data are presented as percentage, mean (SD), or *n* (%) as appropriate.

TAVR, transcatheter aortic valve replacement.

**Table 2:** CT and anatomical parameters of the cohort and subgroup according to primary outcome

Anatomic and CT parameters	Without conduction defect post-TAVR *n* = 54	With conduction defect post-TAVR *n* = 24	Total *n* = 78	*P* value
Tricuspid aortic valve	42 (77.8)	14 (58.3)	56 (71.8)	0.078
Implantation depth below MS (mm)	6.28 (4.21)	7.51 (4.25)	6.66 (4.23)	0.237
Membranous septum (mm)	8.12 (2.87)	6.30 (2.99)	7.56 (3.00)	0.013
Annulus area (mm^2^)	452.57 (86.46)	475.01 (98.31)	459.47 (90.23)	0.314
Calcium score of the aortic valve (Agatston units)	3036.09 (2028.46)	2840.79 (196.85)	1826.90 (2976.24)	0.651
Self-expandable valve	28 (51.9)	12 (50.0)	40 (51.3)	0.880
Type of valve				
Sapien 3	26 (48.1)	12 (50.0)	38 (48.7)	0.836
Corevalve	1 (1.9)	1 (4.2)	2 (2.6)	
Evolut-R	26 (48.1)	10 (41.7)	36 (46.2)	
Lotus	1 (1.9)	1 (4.2)	2 (2.6)	
Valve size (mm)	26.91 (2.12)	27.54 (2.25)	27.10 (2.17)	0.235
Thickness 1 mm below MS (mm)	3.38 (0.52)	2.98 (0.52)	3.25 (0.55)	0.003
Thickness 2 mm below MS (mm)	4.65 (0.78)	4.10 (1.02)	4.48 (0.89)	0.012
Thickness 5 mm below MS (mm)	6.88 (1.03)	6.11 (1.12)	6.65 (1.11)	0.004
Thickness 10 mm below MS (mm)	10.70 (1.55)	9.72 (1.95)	10.40 (1.73)	0.020

Data are presented as percentage, mean (SD), or *n* (%) as appropriate.

MS, membranous septum; TAVR, transcatheter aortic valve replacement; CT, computed tomography.

The composite primary outcome occurred in 24/78 patients (30.8%) as shown in Supplementary material online, *Figure S1*. The indication for PPM implantation was: (1) new complete atrioventricular block in six patients; and (2) Mobitz Type II atrioventricular block in one patient. The PPM was implanted within 2.33 ± 1.03 days following the TAVR.

**Figure 2:**
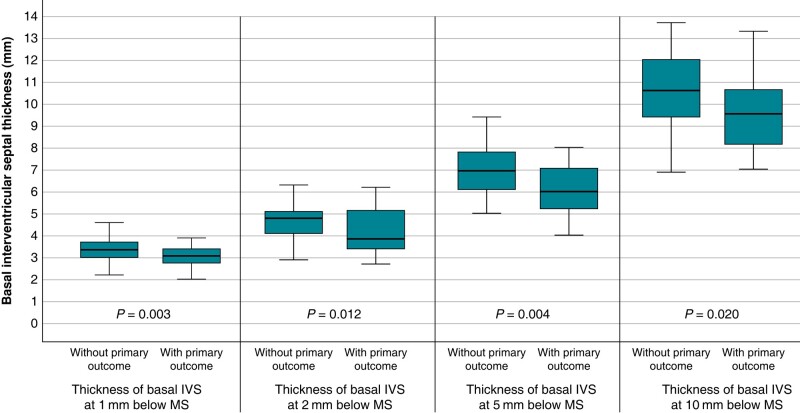
Box plot diagram of IVS thickness in patients with and without primary outcome. Box plot of the thickness of the IVS at 1, 2, 5, and 10 mm below the membranous septum stratified according to the occurrence of conduction disturbances.

Those with the primary outcome were significantly more likely to have a thinner IVS, at all measured levels, than those without the primary outcome as shown in *Figure 2*. Those with the primary outcome were more likely to have a shorter MS (6.30 ± 2.99 mm vs. 8.12 ± 2.87 mm, *P* = 0.013), to be male (66.7% vs. 42.6%, *P* = 0.050) and to have first degree atrioventricular block on the pre-procedural ECG (16.7% vs. 1.9%, *P* = 0.029). Other parameters known as risk factors for conduction defects, including implantation depth, bioprosthetic valve type used, bicuspid aortic valve, and prior right bundle branch block did not differ between patients with and without conduction disturbances (*Tables 1* and *2*).

Decrease in basal IVS thickness, measured at 1 mm below the MS, was associated with increased risk of conduction disturbances following TAVR as shown in *Table 3*, OR = 4.484 for every 1 mm decrease (95% CI: 1.567–12.821, *P* = 0.005). This was also significant at all measured levels of IVS (1, 2, 5, and 10 mm below MS), with ORs (for every 1 mm decrease) of 2.088 (95% CI: 1.152–3.788, *P* = 0.015), 1.996 (95% CI: 1.214; 3.289, *P* = 0.006) and 1.420 (95% CI: 1.047; 1.927, *P* = 0.024) at 2, 5, and 10 mm below the MS, respectively.

**Table 3:** Logistic regression for the risk of the composite outcome according to thickness of basal IVS at levels below membranous septum

	B (SE)	OR	(95% CI)	*P* value
Thickness 1 mm below MS^a^	1.499 (0.535)	4.484	(1.567; 12.821)	0.005
Thickness 2 mm below MS^a^	0.736 (0.303)	2.088	(1.152; 3.788)	0.015
Thickness 5 mm below MS^a^	0.692 (0.254)	1.996	(1.214; 3.289)	0.006
Thickness 10 mm below MS^a^	0.351 (0.156)	1.420	(1.047; 1.927)	0.024

IVS, interventricular septum; MS, membranous septum.

^a^Each for a 1 mm decrease in thickness.

For prediction of primary outcome, a cut-off point of 4 mm muscular IVS thickness at 2 mm level below the MS was the optimal (maximal value of Youden’s index = 0.371) with OR of 4.900 (95% CI: 1.742–13.787, *P* = 0.003) and *c*-statistic of 0.681. The sensitivity and specificity in this cut-off point were 58.3%, and 77.8%, respectively; the positive and negative predictive values were 80.8%, 53.9%, respectively.

Multivariable analysis revealed that pre-procedural IVS thickness with a cut-off point of 4 mm at 2 mm below the MS was the independent predictor of post-procedural conduction disturbance with adjusted OR of 7.387 (95% CI: 2.003–27.244, *P* = 0.003) (*Table 4*). Additional parameters included in the model were: implantations depth, MS, age, complete right bundle branch block, self-expandable valve, and sex. Based on the results of the Wald test of the model, the likelihood ratios for interventricular septal thickness and MS length were 9.019 and 5.611, respectively. Both of these parameters were independent predictors of the primary outcome, however there was no significant interaction between these variables (*P* for interaction 0.832).

**Table 4:** Multivariate analysis of predictive markers for the composite outcome

Parameter	B (SE)	AdjOR	(95% CI)	*P* value
Thickness 2 mm below MS (mm) (<4.0 vs. ≥ 4.0 mm)	2.000 (0.666)	7.387	(2.003; 27.244)	0.003
Implantation depth (1 mm increase)	0.073 (0.089)	1.075	(0.904; 1.280)	0.413
Membranous septum (1 mm decrease)	0.278 (0.117)	1.319	(1.049; 1.661)	0.018
Age (1 year increase)	0.083 (0.041)	1.086	(1.002; 1.177)	0.044
CRBBB	−0.917 (1.065)	0.400	(0.050; 3.222)	0.389
Self-expandable valve	0.267 (0.842)	1.306	(0.251; 6.803)	0.751
Sex (male)	1.205 (0.685)	3.337	(0.872; 12.776)	0.079

CRBBB, complete right bundle branch block; MS, membranous septum.

**Figure 3:**
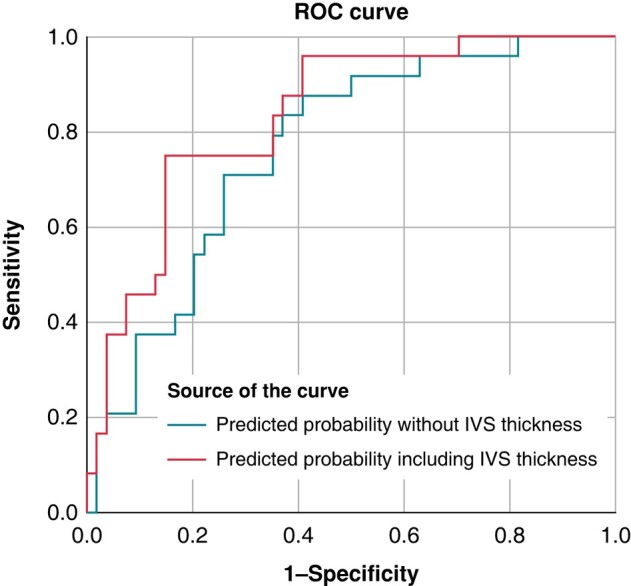
ROC curves for prediction of primary outcome. The ROC curves comparing the additive value of IVS thickness for prediction the primary outcome. The area under the curve (AUC) for the predictive probability of the multivariate model was increased by including IVS thickness. AUC was 0.83 (95% CI: 0.73–0.92) by including IVS thickness and 0.76 (95% CI: 0.65; 0.87) was by excluding IVS thickness. Parameters included in the model were: implantations depth, membranous septum length, age, complete right bundle branch block, self-expandable valve, and sex.

The additional value of this novel marker, thickness of muscular IVS, increased the discriminative ability of the multivariate model from *c*-statistic of 0.76–0.83, with net reclassification improvement of 0.22, as shown in *Figure 3*.

Inter-observer and intra-observer variability and 95% limits (in parenthesis with identical units) for muscular IVS thickness were as follows: for 1 mm below MS 0.03 ± 0.12 (−2.1, 0.27) and 0.02 ± 0.11 (−0.20, 0.24), respectively; for 2 mm below MS 0.06 ± 0.26 (−0.46, 0.58) and 0.03 ± 0.23 (−0.43, 0.49), respectively; for 5 mm below MS 0.02 ± 0.22 (−0.42, 0.46) and 0.01 ± 0.21 (−0.41, 0.43); and for 10 mm below MS 0.01 ± 0.22 (−0.43, 0.45) and 0.05 ± 0.19 (−0.33, 0.43). Interclass correlation coefficient for inter-observer and intra-observer variability at 1 mm below MS was 0.93 and 0.93, respectively; at 2 mm below MS was 0.96 and 0.93, respectively; at 5 mm below MS was 0.99 and 0.95, respectively; and at 10 mm below MS was 0.98 and 0.96, respectively.

## Discussion

**Figure 4:**
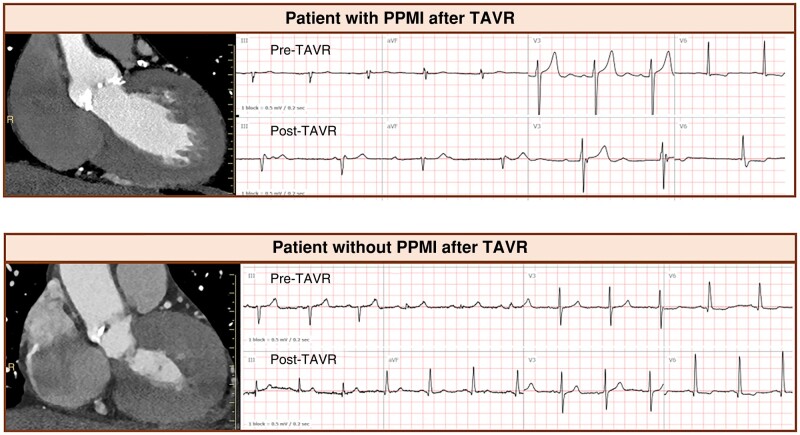
Two representative patients with different basal IVS thickness resulting in different outcomes. (Top) Patient with a relatively thin IVS (IVS was 3.4 mm at 2 mm below MS). Compared with pre-procedural ECG, the post-procedural ECG showed complete atrioventricular block following TAVR. (Bottom) Patients with a relatively thick IVS (IVS was 5.1 mm at 2 mm below the MS) did not develop any conduction disturbances following TAVR.

The main finding of our study is that thickness of the basal muscular IVS, as measured by pre-procedural CT, was predictive of conduction disturbances following TAVR. There was clearly an inverse relationship between a thinner IVS and an increased risk of conduction disturbances following TAVR with an ∼5 times higher risk of conduction disturbances with thickness of <4 mm, measured at the level of 2 mm below the MS. The results demonstrate that basal muscular IVS thickness varies among patients with aortic stenosis referred for TAVR and that this anatomic variation is associated with the risk of conduction disturbances: the thinner the muscular IVS, the higher the risk of conduction abnormalities (*Figure 4*, top). A thicker muscular IVS, on the other hand, may allow protection from device force on the conduction system and therefore prevent conduction abnormalities (*Figure 4*, bottom).

Our findings showed, for the first time, the value of three-dimensional and multiplanar pre-procedural CT for assessing the importance of the basal muscular IVS in the individual patients undergoing TAVR. The key observation strongly supports the pathoanatomic core concept of the present study: the basal IVS thickness emerged as a powerful and independent pre-procedural predictor of conduction disturbances. Indeed, basal IVS thickness remained important even after considering traditional pre-procedural predictors of conduction disturbances.

However, anatomical variations in the location of conduction system in relation to the muscular part of IVS have been described: Kawashima *et al*.^9^ described three different types defining the course of atrioventricular bundle in 105 cadaveric specimens: Type I, the most common course, 46.7%, the atrioventricular bundle ran below a thin layer of ventricular IVS myocardium; Type II, 32.4% of causes, the atrioventricular bundle ran within the interventricular muscle; and Type III, 21% of causes, the atrioventricular bundle is ‘naked’ and located in a subendocardial position without an overlying muscle layer. Our findings are congruent with the anatomical relationship of the conduction system as described above and TAVR landing zone. Our observation suggests that the basal IVS thickness may protect the underlying conduction system from external force of the bioprosthetic device and that those with a thicker IVS have a thicker layer of myocardium overlying the conduction system. These patients may be less likely to have a ‘naked’ exposed atrioventricular bundle. In our study the thickness of the IVS was a significant predictive marker of the primary outcomes together with length of the MS. This reproducible marker can be used for pre-procedural risk assessment and help to individualize periprocedural planning.

An early study in the TAVR era reported that IVS thickness as measured by echocardiography was an independent predictor of PPM^10^; however, these findings were inconsistent.^11,12^ The exact point of measurement, two-dimensional nature of echocardiography, and small patient numbers limited the significance of these findings that were not validated in contemporary cohorts. Tomii *et al*.^13^ examined the degree of basal septal hypertrophy, as evaluated by pre-procedural CT, and found no impact on procedural outcome in patients undergoing TAVR.^13^ To the best of our knowledge, the present study is the first to focus on the predictive value of the IVS thickness itself as a predictive marker of conduction disturbances in patients undergoing TAVR.

### Limitations

The limitations of this study include the relatively small sample size and the retrospective observational nature of the study, and hence, the results are hypothesis generating and need to be validated in larger prospective cohorts. Although we included a relatively small cohort, our study has many inherent strengths including the three-dimensional nature of CT, the precision of measurements, as reflected by low inter- and intra-observer variability, and the fact that the data were taken from a contemporary real-world TAVR cohort. The majority of the conduction disturbances reported in the present study was of LBBB. While LBBB following TAVR may not necessitate PPM implantation, it is not benign in nature and has been shown to be associated with a high incidence of arrhythmic events during follow-up.^14^ Our findings add to other known risk factors for CD following TAVR. The optimal strategy of monitoring for conduction disturbance following TAVR is uncertain and should take multiple, and no single, patient-specific factors into account.^15,16^

## Conclusions

The thickness of the basal muscular part of the IVS, as assessed by CT, may act as an ‘anatomical cushion’ protecting to some degree the underlying conduction system from external force of the implanted bioprosthetic valve. Interventricular septum thickness is a novel predictive marker for the risk of conduction disturbances in patients undergoing TAVR.

## Supplementary Material

euae113_Supplementary_Data

## Data Availability

The data that support the findings of this study are available from the corresponding author, N.S.P., upon reasonable request.
