# Supplementary figures and images for: Interventricular septal thickness on cardiac computed tomography as a novel risk factor for conduction disturbances in patients undergoing transcatheter aortic valve replacement
Source: Europace. 2024 May 1;26(5):euae113. doi: 10.1093/europace/euae113 (PMC11094757; doi:10.1093/europace/euae113)

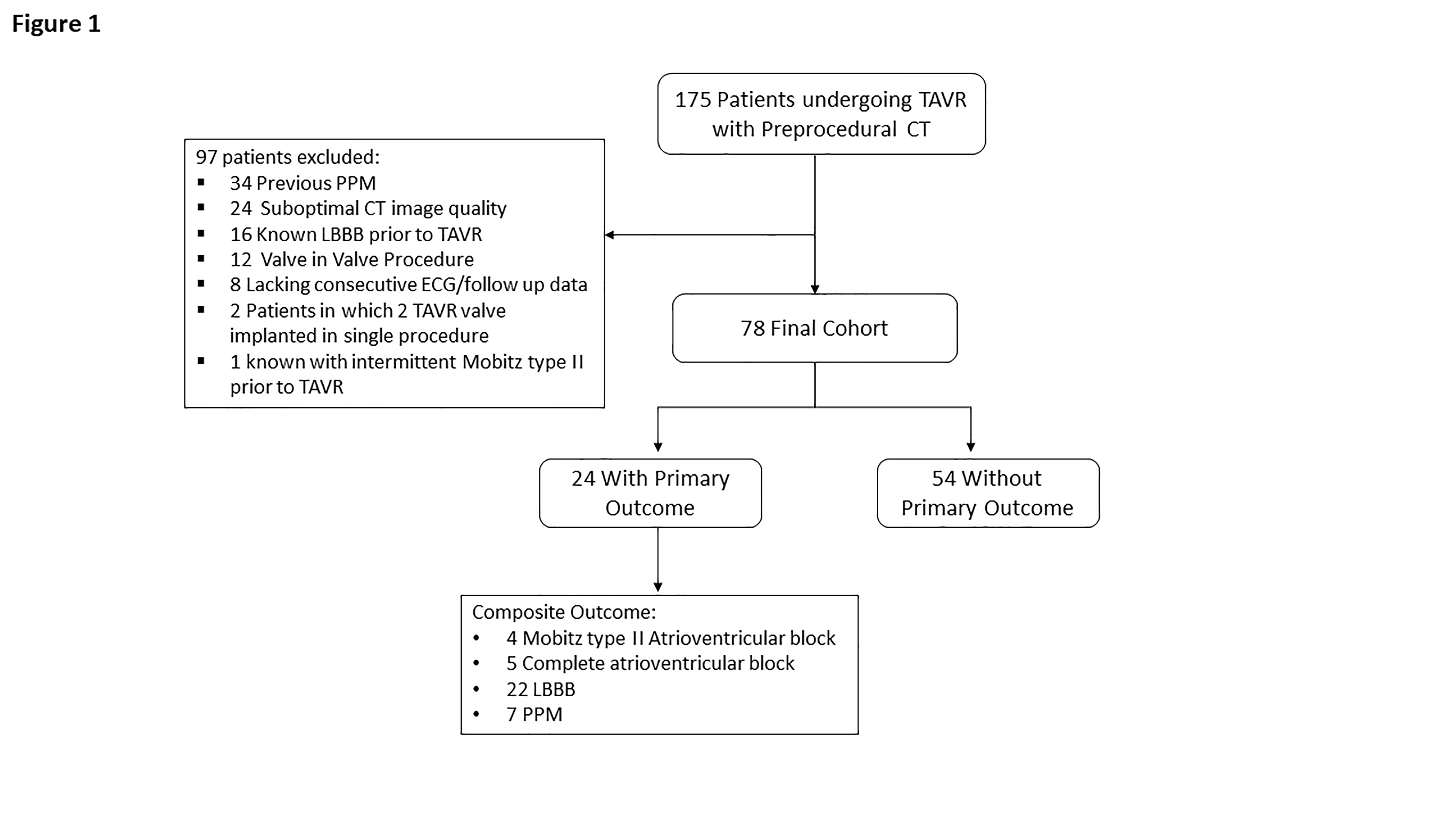

Supplement: euae113_Supplementary_Data [file euae113_supplementary_data.zip › Supplementary Figure 1.tif]
